# Supplementary material for: A conserved major facilitator superfamily member orchestrates a subset of O-glycosylation to aid macrophage tissue invasion
Source: eLife. 2019 Mar 26;8:e41801. doi: 10.7554/eLife.41801 (PMC6435326; doi:10.7554/eLife.41801)
Supplement: Supplementary file 3. — Columns list the gene name for the receptor, its reported function, what kind of glycosylation we identified to be present on the receptors in the wild type sample, and what kind of glycosylation change we observed in the mrva3102 mutant. [file elife-41801-supp3.docx]

| **Receptor** | **Function** | **Glycosylation** | **Changes in *mrva3102*** |
| --- | --- | --- | --- |
| Babo | Activin signaling | 2 glyco sites, T antigen only | no |
| Boi | Regulation of Hh-dependent processes | 3 glyco sites, Tn antigen only | no |
| CG12121 | Unknown | 3 glyco sites, Tn antigen only | no |
| CG15765 | Carbohydrate binding, nervous system development | 1 glycosite, T antigen | no |
| CG5888 | Unknown | 1 glyco site, T or Tn antigen | no |
| CG9095 | Carbohydrate binding | 1 glyco site, Tn antigen | no |
| Cirl | Calcium independent receptor for a-latrotoxin, adult locomotory behavior | 1 glyco site, T or Tn antigen | no |
| Crb | epithelial morphogenesis, apico- basal cell polarity, negative regulator of Notch activity | 1 glyco site, Tn antigen | no |
| Dg | non-integrin ECM receptor, connects ECM to the actin cytoskeleton | 1 glycosite, T or Tn antigen | no |
| Drl | axon guidance through Wnt5 | 1 glyco site, T or Tn antigen | no |
| Hbs | Muscle cell fusion | 2 glycosites, T and Tn antigen | no |
| Hmu | Hydrolase activity | 15 glycosites, both T and Tn antigen | Tn inc. |
| LpR1 | Regulation of immune responses | 2 glycosites, Tn antigen | Tn inc. |
| LpR2 | Cellular uptake of neutral lipids | 3 sites, T and Tn antigen | T & Tn inc. |
| LRP1 | LDL receptor, works with megalin | 4 glycosites, T and Tn antigen | no |
| Mgl | Lipid regulation | 2 glycosites, Tn antigen | Tn dec. |
| Mthl5 | GPCR, heart morphogenesis | 1 glyco site, T or Tn antigen | no |
| NimB2 | Defense response to bacterium | 1 glycosite, Tn antigen | no |
| NimC4 | Recognition and engulfment of apoptotic cells during development | 1 glycosite, T or Tn antigen | no |
| Nrx-IV | Septate junction formation, glial neural interaction | 1 glyco site, Tn antigen | no |

| PlexB | Axon guidance | 1 glyco site, Tn antigen | no |
| --- | --- | --- | --- |
| Put | Dpp signaling | 5 glyco sites, T and Tn antigen | T&Tn dec. |
| Sas | Pathfinding, glial neuron interaction |  | T dec. |
| Sdc | Robo neural pathfinding, synapse at neuromascular junction | 1 glyco site, Tn antigen | no |
| Sema-1b | Neural pathfinding | 1 glyco site, Tn antigen | no |
| Sli | Neural pathfinding, robo interaction | 2 glyco sites, T and Tn antigen | T & Tn inc. |
| Sr-CII | Scavenger receptor, immune response | 6 glyco sites, T and Tn antigen | no |
| Syb | Synaptic vesicle, SNAP receptor activity | 1 glyco site, T or Tn antigen | no |
| Tequila | Scavenger receptor, serine protease, glucose homeostasis, long and short term memory | 5 glycosite, Tn antigen | no |
| Unc-5 | Neural pathfinding, netrin receptor | 1 glycosite, Tn antigen | no |
| Verm | Cuticle development and tracheal tube size control | 1 glycosite, T or Tn antigen | T & Tn inc. |
